# FIRST BRAZILIAN CONSENSUS ON CHOLANGIOPANCREATOSCOPY BY THE BRAZILIAN SOCIETY OF DIGESTIVE ENDOSCOPY (SOBED)

**DOI:** 10.1590/S0004-2803.24612025-057

**Published:** 2026-01-09

**Authors:** Tomazo Antônio Prince FRANZINI, Bruno da Costa MARTINS, Alexandre Moraes BESTETTI, Carlos Henrique Barros AMARAL, Cláudio Rogério SOLAK, Djalma Ernesto COELHO, Eduardo Guimarães Hourneaux DE MOURA, Eduardo Michels OPPITZ, Fernanda Prata MARTINS, Flavio Hayato EJIMA, Gerson Cesar BRASIL, Gustavo Andrade DE PAULO, Gustavo de Oliveira LUZ, Leonardo VALLINOTO, Marcos Eduardo Lera DOS SANTOS, Rafael William NODA, Raquel Canzi Almada DE SOUZA, Renato Luz CARVALHO, Rodrigo Roda Rodrigues SILVA, Tiago CARDOSO, Victor Rossi BASTOS, Júlia LIMA, Ruth Bartelli GRIGOLON, Vicky Nogueira PILEGGI, Fauze MALUF-FILHO

**Affiliations:** 1 Hospital das Clínicas da Universidade de São Paulo, Unidade de Endoscopia Gastrointestinal, São Paulo, SP, Brasil.; 2 Instituto do Câncer da Universidade de São Paulo, Departamento de Gastroenterologia, Divisão de Endoscopia, São Paulo, SP, Brasil.; 3 Santa Casa de Misericórdia de Maceió, Serviço de Endoscopia Digestiva, Maceió, AL, Brasil.; 4 Hospital Universitário de Ponta Grossa, Departamento de Endoscopia, Ponta Grossa, PR, Brasil.; 5 Hospital Américas Medical City, Serviço de Gastroenterologia e Endoscopia, Coordenador Médico, Rio de Janeiro, RJ, Brasil.; 6 Hospital das Clínicas da FMUSP, Serviço de Endoscopia Gastrointestinal e Broncoesofagoscopia, São Paulo, SP, Brasil.; 7 Hospital Moinhos de Vento, Porto Alegre, RS, Brasil.; 8 Hospital Israelita Albert Einstein, São Paulo, SP, Brasil.; 9 Instituto Hospital de Base do Distrito Federal, Brasília, DF, Brasil.; 10 Hospital Universitário Oswaldo Cruz da Universidade de Pernambuco, Serviço de Endoscopia Digestiva, Recife, PE, Brasil.; 11 Hospital Israelita Albert Einstein, Serviço de Endoscopia, São Paulo, SP, Brasil.; 12 Hospital Nossa Senhora das Graças, Unidade de Endoscopia, Curitiba, PR, Brasil.; 13 Universidade Federal do Paraná, Curitiba, PR, Brasil.; 14 Hospital do Servidor Público Estadual (IAMSPE) - Rede Santa Catarina, Diretor do Serviço de Endoscopia, São Paulo, SP, Brasil.; 15 Hospital das Clínicas da Universidade Federal de Minas Gerais, Instituto Alfa de Gastroenterologia; Rede Mater Dei de Saúde, Belo Horizonte, MG, Brasil.; 16 Fundação Hospital Adriano Jorge, Manaus, AM, Brasil.; 17 Hospital São Rafael, Salvador, BA, Brasil.; 18 Oracle Life Sciences, São Paulo, SP, Brasil.; 19 Faculdade de Medicina da Universidade de São Paulo, Departamento de Gastroenterologia, Instituto do Câncer do Estado de São Paulo, Pesquisador CNPq, São Paulo, SP, Brasil.

**Keywords:** Cholangiopancreatoscopy, single-operator peroral cholangioscopy, indeterminate biliary strictures, primary sclerosing cholangitis, peroral pancreatoscopy, consensus, Colangiopancreatoscopia, colangioscopia peroral de operador único, estenoses biliares indeterminadas, colangite esclerosante primária, pancreatoscopia peroral, consenso

## Abstract

**Background:**

Despite the widespread adoption of single-operator cholangiopancreatoscopy for the management of biliary and pancreatic diseases, this method was recently introduced in Brazil. This study aimed to develop a Brazilian consensus for the diagnostic and therapeutic use of single-operator cholangiopancreatoscopy for the management of pancreatobiliary diseases.

**Methods:**

A working group from the Brazilian Society of Digestive Endoscopy (SOBED) specialists, using a Delphi methodology, formulated statements across six topics: indeterminate biliary strictures, complex biliary stones, primary sclerosing cholangitis, peroral pancreatoscopy, post-liver transplantation, and antibiotic prophylaxis. Eighteen experts evaluated these statements using an anonymous electronic voting system. Consensus was defined as ≥80% agreement.

**Results:**

Eighteen statements were formulated and voted on by a panel of experts to reach a consensus. The panel consisted of 18 endoscopy specialists from various regions of Brazil, with an average of 20.4 years of experience. Consensus was achieved on all statements in the first round.

**Conclusion:**

These recommendations establish the role of cholangiopancreatoscopy in diagnostic and therapeutic for indeterminate biliary strictures, complex biliary stones, primary sclerosing cholangitis, peroral pancreatoscopy, post-liver transplantation, and antibiotic prophylaxis, providing a background for its standardized use in Brazil.

## HIGHLIGHTS

•This consensus establishes evidence-based recommendations for the diagnostic and therapeutic use of single-operator cholangiopancreatoscopy in Brazil.

•Eighteen experts reached agreement on 18 statements covering indeterminate biliary strictures, complex biliary stones, primary sclerosing cholangitis, peroral pancreatoscopy, post-liver transplantation, and antibiotic prophylaxis.

•These recommendations provide a framework to standardize cholangiopancreatoscopy practice and improve outcomes for patients with pancreatobiliary diseases.

## RESUMO

**Contexto:**

Apesar da ampla adoção da colangiopancreatoscopia de operador único para o manejo das doenças biliares e pancreáticas, esse método foi introduzido recentemente no Brasil. Este estudo teve como objetivo desenvolver um consenso brasileiro para o uso diagnóstico e terapêutico da colangiopancreatoscopia de operador único no manejo das doenças pancreatobiliares.

**Métodos:**

Um grupo de trabalho da Sociedade Brasileira de Endoscopia Digestiva (SOBED), utilizando a metodologia Delphi, formulou recomendações em seis tópicos: estenoses biliares indeterminadas, cálculos biliares complexos, colangite esclerosante primária, pancreatoscopia peroral, pós-transplante hepático e profilaxia antibiótica. Dezoito especialistas avaliaram essas recomendações por meio de um sistema eletrônico de votação anônima. O consenso foi definido como ≥80% de concordância.

**Resultados:**

Foram formuladas dezoito recomendações, avaliadas e votadas por um painel de especialistas até alcançar consenso. O painel foi composto por 18 endoscopistas de diferentes regiões do Brasil, com média de 20,4 anos de experiência. O consenso foi alcançado em todas as recomendações já na primeira rodada.

**Conclusão:**

Essas recomendações estabelecem o papel da colangiopancreatoscopia no diagnóstico e tratamento das estenoses biliares indeterminadas, cálculos biliares complexos, colangite esclerosante primária, pancreatoscopia peroral, pós-transplante hepático e profilaxia antibiótica, fornecendo subsídios para seu uso padronizado no Brasil.

## INTRODUCTION

Biliary and pancreatic diseases present significant challenges in gastroenterology, often requiring advanced diagnostic and therapeutic interventions. While established techniques such as endoscopic retrograde cholangiopancreatography (ERCP) are effective for diagnosing and managing many biliary conditions, a subset of cases remains difficult to address^1^. A major challenge lies in diagnosing the etiology of biliary strictures, which is crucial for determining patient management and prognosis. ERCP, in conjunction with biopsy and/or brushing, often provides limited diagnostic sensitivity, determining a complex task to resolve. Additionally, the management of difficult to remove stones, especially when ERCP with mechanical lithotripsy or balloon papillary dilation fails, poses further clinical challenges^2^.

In this context, cholangiopancreatoscopy has emerged as a promising solution. In 2015, the National Institute for Health and Care Excellence (NICE) published a health technology assessment of the SOC-SpyGlass system for the diagnostic and therapeutic management of biliary diseases, recommending its use when standard techniques fail or are deemed inappropriate^3^. The 2019 European Society of Gastrointestinal Endoscopy (ESGE) guidelines endorsed cholangioscopy-assisted intraluminal lithotripsy (electrohydraulic or laser) as an effective and safe approach for treating difficult bile duct stones^4^. Furthermore, recent advancements in single operator per-oral cholangioscopy (SOC) have revolutionized the management of biliary and pancreatic diseases. With improved imaging resolution, SOC has enhanced the ability to visualize biliary and pancreatic structures in greater detail. This innovation allows for more accurate assessment of stricture morphology and facilitates targeted biopsies, thereby increasing diagnostic sensitivity and enabling more precise treatment strategies for these complex conditions.

Despite these advancements, the precise role of cholangiopancreatoscopy in the overall management of biliary and pancreatic diseases remains unclear. Currently, there are no established guidelines on the application of cholangiopancreatoscopy for these conditions. To address this gap, this study aims to develop consensus-based clinical guidelines for the diagnostic and therapeutic use of cholangiopancreatoscopy in the management of biliary and pancreatic diseases in Brazil.

## METHODS

### Study design

This study used the Delphi methodology to achieve consensus among experts on the applicability of cholangiopancreatoscopy in the diagnosis and treatment of biliary and pancreatic diseases, with the goal of developing consensus-based clinical recommendations for gastrointestinal disease management. The Delphi method was selected as a structured communication technique, ideal for situations where uncertainty exists, or scientific evidence is limited.

### Selection of experts

Experts were selected based on their recognized expertise and experience in the field. The panel comprised 18 specialists in endoscopy from diverse regions of Brazil, regardless of their practice setting (public or private). This ensured a broad representation of views and experiences in the field.

### Questionnaire development

A working group composed of four medical specialists (TAPF, BCM, AMB, and FMF), members of the Brazilian Society of Digestive Endoscopy (SOBED), and three experts in Delphi methodology (RBG, VNP, and JL) from Oracle Life Sciences, met over a period of three months to review the available literature and develop the questionnaire. To identify the available literature, search strategies were developed in the MEDLINE (via PubMed), LILACS (via BVS), and Cochrane databases in July 2024. Only studies published after 2007 were included, the year in which SOC with direct visualization systems was first described. Table 1 presents the complete search strategies.

**TABLE 1:** Search strategies used in the MEDLINE (via PubMed), LILACS (via BVS), and Cochrane databases.

Database	Search terms	Results
MEDLINE (PubMed)	(“Bile Duct Diseases”[Mesh] OR “Bile Duct Neoplasms”[Mesh] OR “Gallstones”[Mesh] OR “Bile Ducts”[Mesh]) OR (Indeterminate Biliary Strictures) OR (Biliary Strictures) OR (“Cholangitis, Sclerosing”[Mesh]) OR (Primary Sclerosing Cholangitis) OR (Cholangiocarcinoma) OR (Intraductal lithotripsy) OR (Pancreatic stones) OR (“Pancreatic Intraductal Neoplasms”[Mesh]) OR (Post-Liver Transplant Biliary) OR (post-transplant biliary strictures) OR (biliary strictures post liver transplantation) OR (biliary strictures post-liver transplantation) OR (Complex Biliary Stones) AND ((((cholangioscop*) OR (Single-Operator Cholangioscopy)) OR (SOC)) OR (Pancreatoscop*) OR (SpyGlass)) **AND** (((((((((Diagnostic accuracy[Title/Abstract]) OR (Sensitivity[Title/Abstract])) OR (Specificity[Title/Abstract])) OR (Adverse events[Title/Abstract])) OR (Complications[Title/Abstract])) OR (Preoperative planning[Title/Abstract])) OR ((surger*[Title/Abstract] OR surgic*[Title/Abstract]) **AND** (therapy[Title/Abstract] OR treatment[Title/Abstract] OR manage*[Title/Abstract])) ) OR (“Diagnosis”[Mesh] OR “Diagnostic Techniques and Procedures”[Mesh])) OR (“Surgical Procedures, Operative”[Mesh] OR “surgery” [Subheading] OR “General Surgery”[Mesh])) OR (Guidewire-assisted placement) OR (Guidewire placement) OR (stricture resolution) OR (percutaneous biliary intervention) OR (Stone clearance)	1,149
LILACS (via BVS)	“Bile Duct Diseases” OR “Bile Duct Neoplasms” OR “Gallstones” OR “Bile Ducts” OR “Indeterminate Biliary Strictures” OR “Biliary Strictures” OR “Cholangitis, Sclerosing” OR “Primary Sclerosing Cholangitis” OR “Cholangiocarcinoma” OR “Intraductal lithotripsy” OR “Pancreatic stones” OR “Pancreatic Intraductal Neoplasms” OR “Post-Liver Transplant Biliary” OR “post-transplant biliary strictures” OR “biliary strictures post liver transplantation” OR “biliary strictures post-liver transplantation” OR “Complex Biliary Stones” **AND** “cholangioscop*” OR “Single-Operator Cholangioscopy” OR “SOC” OR “Pancreatoscop*” OR “SpyGlass” **AND** “Diagnostic accuracy” OR “Sensitivity” OR “Specificity” OR “Adverse events” OR “Complications” OR “Preoperative planning” OR (“surger*” OR “surgic*” AND “therapy” OR “treatment” OR “manage*”) OR “Diagnosis” OR “Diagnostic Techniques and Procedures” OR “Surgical Procedures, Operative” OR “surgery” OR “General Surgery” OR “Guidewire-assisted placement” OR “Guidewire placement” OR “stricture resolution” OR “percutaneous biliary intervention” OR “Stone clearance”	7
COCHRANE	“Bile Duct Diseases” OR “Bile Duct Neoplasms” OR “Gallstones” OR “Bile Ducts” OR “Indeterminate Biliary Strictures” OR “Biliary Strictures” OR “Cholangitis, Sclerosing” OR “Primary Sclerosing Cholangitis” OR “Cholangiocarcinoma” OR “Intraductal lithotripsy” OR “Pancreatic stones” OR “Pancreatic Intraductal Neoplasms” OR “Post-Liver Transplant Biliary” OR “post-transplant biliary strictures” OR “biliary strictures post liver transplantation” OR “biliary strictures post-liver transplantation” OR “Complex Biliary Stones” **AND** “cholangioscopy” OR “Single-Operator Cholangioscopy” OR “SOC” OR “Pancreatoscopy” OR “SpyGlass” AND “Diagnostic accuracy” OR “Sensitivity” OR “Specificity” OR “Adverse events” OR “Complications” OR “Preoperative planning” OR (“surgery” OR “surgical” AND “therapy” OR “treatment” OR “management”) OR “Diagnosis” OR “Diagnostic Techniques and Procedures” OR “Surgical Procedures, Operative” OR “surgery” OR “General Surgery” OR “Guidewire-assisted placement” OR “Guidewire placement” OR “stricture resolution” OR “percutaneous biliary intervention” OR “Stone clearance”	61

### Delphi procedure

The consensus was structured into the following areas, with the corresponding number of statements:

- Indeterminate biliary strictures - 6 statements
- Complex biliary stones - 5 statements
- Primary sclerosing cholangitis - 1 statement
- Peroral pancreatoscopy - 4 statements
- Post-liver transplantation - 1 statement
- Antibiotic prophylaxis recommendation - 1 statement

Eighteen experts participated in the Delphi process, which was conducted through an anonymous electronic voting system. Each statement was accompanied by a list of references supporting it.

The Delphi panel was initially planned to be conducted in three steps. However, since consensus was reached on all statements in the first round, no additional rounds were necessary. The first-round questionnaire was distributed to the experts via a link, and they indicated their level of agreement with each statement using the 5-point Likert scale. Responses were aggregated, and the distribution of scores was analyzed for each item.

The 5-point Likert scale was used to collect responses:

- 1=strongly disagree
- 2=disagree
- 3=neutral or indifferent
- 4=agree
- 5=strongly agree

### Consensus criteria and Level of evidence

Consensus was defined as at least 80% agreement among the experts, meaning that 80% of participants would indicate “Agree” or “Strongly Agree” (scores 4 or 5 on the Likert scale) for a given statement. The Oxford CEBM (centre for evidence based medicine) levels of evidence were used in this guideline were classified according to a hierarchy based on study design^5^. This classification system provides a structured approach to evaluating the level of evidence, ranging from systematic reviews and randomized controlled trials (RCTs) to observational studies and expert opinions. Level IA corresponds to evidence derived from meta-analyses of RCTs, while level IB includes data from at least one RCT or meta-analyses combining RCTs and non-RCTs. Levels IIA and IIB refer to controlled studies without randomization and other quasi-experimental studies, respectively. Level III includes evidence from nonexperimental descriptive studies such as case-control, correlation, or comparative studies. Finally, level IV is based on expert committee reports, clinical experience, or authoritative opinions. The evidence used in this guideline was classified using the scheme^6^ of Table 2.

**TABLE 2:** Classification of Level of evidence.

Evidence level description	
IA	Evidence from meta-analysis of RCTs
IB	Evidence from at least 1 RCT or meta-analysis using RCTs and non-RCTs
IIA	Evidence from at least 1 controlled study without randomization
IIB	Evidence from at least 1 other type of quasi-experimental study
III	Evidence from nonexperimental descriptive studies, such as comparative studies, correlation studies and case-control studies
IV	Evidence from expert committee reports or opinions or clinical experience of respected authorities, or both

## RESULTS

### Characterization of expert sample

A total of 18 experts participated in the study. Among them, 16 (89%) were male, and the average age was 50 years. The majority of the experts worked in both the private and public healthcare systems (61%, n=11), while 33% (n=6) worked exclusively in the private sector, and 6% (n=1) worked exclusively in the public sector. The average number of years since completing their specialization in endoscopy was 20.4 years (range 10-34 years). Regarding the annual number of cholangiopancreatoscopy procedures performed, 39% (n=7) conducted fewer than 10 procedures, 44% (n=8) performed between 10 and 20 procedures, and 17% (n=3) performed 20 or more. As for the number of ERCP procedures conducted annually, no expert performed fewer than 50; 6% (n=1) performed between 50 and 100, 33% (n=6) performed between 100 and 200, and 61% (n=11) performed 200 or more. In terms of geographic distribution, 44% (n=8) of the experts were based in the Southeast, 22% (n=4) in the South, 17% (n=3) in the Northeast, 11% (n=2) in the North, and 6% (n=1) in the Midwest.

### Indeterminate biliary strictures

Recommendation 1: Indeterminate biliary stricture refers to a condition without a defined etiology, characterized by inconclusive or negative findings on imaging and tissue sampling.

(Level of evidence: III; rate of agreement: 100%).

Indeterminate biliary strictures involve narrowing of the intrahepatic or extrahepatic bile ducts in the absence of detectable masses or lesions on imaging modalities such as computed tomography (CT) or magnetic resonance imaging (MRI), and without evident clinical causes such as recent trauma or surgical interventions. In such cases, conventional diagnostic methods fail to establish a definitive diagnosis, with the primary goal being to identify or exclude malignancy^7^ ^,^ ^8^. Since approximately 50-70% of indeterminate biliary strictures are of malignant origin^9^ ^,^ ^10^, it is crucial to achieve an early and accurate diagnosis, particularly to identify malignancies at an early stage, as this significantly improves survival rates. Conversely, misdiagnosis can have equally severe consequences, potentially leading to unnecessary surgeries in patients with benign conditions^10^ ^,^ ^11^.

Recommendation 2: direct visualization using SOC, combined with guided biopsy, should be the first choice for the etiological evaluation of indeterminate biliary strictures. The combination of these techniques significantly improves diagnostic sensitivity compared to endoscopic retrograde cholangiopancreatography (ERCP) with cytologic brushing and/or transpapillary biopsy.

(Level of evidence: IB; rate of agreement: 94.5%).

Differentiating between benign and malignant biliary strictures remains a challenge in clinical practice. ERCP with cytologic brushing and/or transpapillary biopsy is the most commonly employed technique for this purpose. However, the sensitivity of ERCP for evaluating indeterminate biliary strictures is below 60%^12^.

Only one randomized clinical trial has compared the diagnostic efficacy of SOC-guided visualization and biopsy versus standard ERCP with standard brushing in patients with indeterminate biliary strictures^13^. SOC demonstrated significantly higher sensitivity for both tissue-sampling-based diagnosis (68.2% vs 21.4%; *P*<0.01) and endoscopic visual assessment (95.5% vs 66.7%; *P*= 0.02), with a comparable adverse event rate between groups^13^.

Several observational studies have evaluated the efficacy and safety of SOC-based visual assessment and tissue sampling for diagnosing indeterminate biliary strictures, using histopathological analysis of surgical specimens, autopsy findings, or long-term clinical follow-up as the reference standard^14^ ^-^ ^20^. A systematic review with meta-analysis published in 2020 synthesized these studies^21^. The review included 11 studies - 5 prospective cohorts, 6 retrospective cohorts, and 2 randomized clinical trials - comprising a total of 323 patients. SOC-guided biopsy showed a pooled sensitivity of 0.74 (95%CI: 0.67-0.80) and a specificity of 0.98 (95%CI: 0.95-1.00), with an area under the curve (AUC) of 0.948. For direct visualization via SOC, the sensitivity was 0.95 (95%CI: 0.91-0.97) and the specificity 0.92 (95%CI: 0.88-0.95), with an AUC of 0.977.

The pooled adverse event rate for SOC was 7% (95%CI: 3-12%, I²=81%). The primary complications included post-ERCP pancreatitis, cholangitis, bleeding, and perforation, with acute cholangitis being the most common, occurring in 1.8% of cases (95%CI: 0.8-3.1%; I²=76.4%). No severe complications or procedure-related mortality were reported for SOC.

Recommendation 3: the efficacy of SOC may be limited in the evaluation of distal biliary strictures due to inherent technical challenges.

(Level of evidence: III; rate of agreement: 94.5%).

In some cases, the effectiveness of SOC in assessing distal biliary strictures can be compromised due to technical difficulties. Kurihara and colleagues demonstrated that tissue samples obtained from lesions in the distal common bile duct exhibited the lowest adequacy compared to other locations (70% vs 79-86%; *P* value not reported)^22^. Moreover, in a multivariate analysis conducted by Onoyama and colleagues, the adequacy of SOC-guided biopsy samples from distal biliary strictures was significantly reduced (OR=0.32; 95%CI: 0.14-0.74) compared to those from the proximal bile duct^23^.

Recommendation 4: the accuracy of SOC is directly dependent on the experience, training, and skill of the operator.

(Level of evidence: III; rate of agreement: 100%).

SOC has significantly advanced the field by enabling direct visualization of the biliary system and strictures. The publication of standardized classification systems for SOC visual analysis has markedly improved interobserver agreement. Milluzzo et al. conducted a study evaluating interobserver agreement in 29 SOC video assessments, demonstrating an almost perfect agreement for the final diagnosis (kappa 0.871; agreement rate of 93.1%; *P*<0.001)^17^. However, overall accuracy was higher among specialists compared to trainees (73.6% vs 64.4%, respectively; *P* value not reported)^17^. A retrospective study by Jang et al. showed, through multivariate analysis, that experience with the technique - defined as performing more than 50 procedures - was significantly associated with improvements in both accurate visual interpretation (OR 16.6; 95%CI: 27.5-100.3) and successful tissue sampling (OR 11.29; 95%CI: 1.89-67.26)^14^. Therefore, the success rates of SOC are higher when performed by experienced endoscopists.

Recommendation 5: for the diagnosis of indeterminate biliary strictures using SOC, at least three targeted biopsies or the presence of an onsite pathologist is recommended. Collecting an adequate number of samples is essential to improve diagnostic accuracy and reduce the likelihood of inconclusive or false-negative results.

(Level of evidence: IB; rate of agreement: 94%)

The diagnostic accuracy of SOC is directly correlated with the number of biopsies performed; however, the reviewed studies revealed considerable variability in the number of biopsies, ranging from 2 to 6, without standardized protocols. A systematic review demonstrated that sensitivity for the etiological assessment of indeterminate biliary strictures was higher in studies performing more than two biopsies (0.75; 95%CI: 0.68-0.81) compared to those with fewer than two biopsies (0.69; 95%CI: 0.49-0.85)^21^. These findings highlight the importance of collecting an adequate number of samples for accurate diagnosis. Bang et al. conducted a randomized clinical trial in patients with indeterminate biliary strictures, comparing two groups: one with onsite pathologist evaluation (n=32) and another with offsite sample processing (n=30). In the offsite group, seven fragments were collected, with three placed in one container and four in another. Diagnostic accuracy was comparable between the groups (onsite 87.5% vs offsite 90%; *P*=0.99). Moreover, no significant difference in diagnostic accuracy was observed when comparing the collection of three versus four biopsies within the offsite cohort (*P*=0.999). These findings suggest that while an increased number of biopsies is necessary when samples are processed offsite, collecting at least three targeted biopsies ensures diagnostic precision^24^.

Recommendation 6: the use of SOC directly impacts the management of patients with indeterminate biliary strictures, guiding the need for intervention, preventing unnecessary procedures, or determining the appropriate extent of surgery.

(Level of evidence: III; rate of agreement: 100%).

Studies such as that by Almadi et al. have shown that SOC influenced the treatment of 86.2% of patients (249 of 289), either by identifying the cause of biliary strictures in 50.9% of cases or by clarifying filling defects and biliary dilations in 37.4%^25^. Furthermore, SOC detected biliary stones missed by other evaluation methods in 1.7% of patients. During the same ERCP session in which cholangioscopy was performed, 29.4% of patients received biliary stents. Surgical intervention was avoided in 22.2% of cases, while 19.0% of patients were referred for surgery, and 5.19% were directed to combined chemoradiotherapy. Another pivotal study by Prat et al. demonstrated that the management plan was altered in 58.3% of patients with indeterminate biliary strictures after SOC evaluation^26^. While the initially planned surgery proceeded in 20 out of 48 patients, the management strategy shifted to a conservative approach in 26 cases. Two patients required more aggressive treatments, with one referred for surgery and another for chemotherapy.

Tyberg et al. investigated the role of SOC in mapping pancreatobiliary neoplasms and assessing disease extent before surgical resection. The study aimed to determine whether SOC could modify pre-established surgical plans. The findings revealed that SOC altered surgical plans in 32 out of 105 patients (30.5%) with bile duct involvement. Among these, 6 patients (5%) underwent less extensive surgeries than initially planned. Surgery was avoided in 26 patients (25%): 14 were diagnosed with benign diseases, and 12 had more extensive disease, rendering them ineligible for surgery. The study also reported an 86% correlation between endoscopic findings and histological results, indicating that SOC effectively assessed disease extent in the bile duct and contributed to more precise surgical decision-making^27^.

Deprez and colleagues assessed the clinical and economic impact of SOC compared to ERCP in the diagnosis of indeterminate biliary strictures. The analysis included data from 49 patients treated at Belgian hospitals and showed that the use of SOC led to a 31% reduction in the number of procedures and an estimated cost saving of €12,990 per year from a hospital perspective. Although the cost of acquiring SOC equipment was a significant component, this was balanced by reduced hospitalization expenses and improved diagnostic yield through optically guided biopsies. Overall, SOC proved to be a more efficient and cost-effective strategy for diagnosing indeterminate biliary strictures^28^.

### Complex biliary stones (difficult to remove stones, intrahepatic stones, stones in cystic duct remnants)

Recommendation 1: difficult-to-remove stones, also referred to as complex or challenging stones, are defined as those with large dimensions (>15 mm), multiple stones, stones associated with Mirizzi syndrome, disproportion between the stone size and the distal bile duct diameter (greater than 2 mm), stones located proximal to a stricture, or stones with unusual shape (e.g., barrel-shaped) or location (e.g., cystic duct or intrahepatic).

(Level of evidence: IA; rate of agreement: 100%).

A systematic review included in this analysis defines difficult-to-remove stones as large, multiple, intrahepatic, cystic duct, or impacted stones; stones associated with Mirizzi syndrome; or stones located proximal to a stricture^29^. Similarly, clinical trials included in the review reported comparable definitions, with some defining large stones as those >10 mm^30^ and others as >15 mm^31^ ^,^ ^32^. Retrospective^33^ and prospective^34^ ^,^ ^35^ studies also described difficult stones based on previous procedural failure, size >15 mm, multiple stones, intrahepatic/cystic duct location, barrel-shaped morphology, or impaction^33^.

Recommendation 2: SOC with electrohydraulic lithotripsy (EHL) or laser lithotripsy (LL) is recommended for the management of difficult-to-remove stones in cases where ERCP with mechanical lithotripsy or balloon papillary dilation has failed.

(Level of evidence: IB; rate of agreement: 100%)

Buxbaum et al. conducted a randomized clinical trial comparing cholangioscopy-guided laser lithotripsy with conventional therapy in patients with bile duct stones >1 cm. Successful bile duct clearance was achieved in 93% (39/42) of patients undergoing cholangioscopy, compared to 67% (12/18) of those receiving conventional therapy (*P*=0.009). The adjusted odds ratio for prior ERCP failure was 8.0 (95%CI: 1.6-40.2)^30^.

Another randomized trial included 32 patients with large stones in the common hepatic duct or bile duct with disproportionate stone-to-duct diameter and prior failed ERCP attempts with conventional sphincterotomy. Patients were randomized to mechanical lithotripsy or laser lithotripsy guided by cholangioscopy. Mechanical lithotripsy had a lower success rate (63% vs 100%; *P*<0.01) and resulted in higher radiation exposure (*P*=0.04)^31^.

Additionally, Bang et al. performed a randomized trial on patients with incomplete bile duct clearance following conventional ERCP. The SOC + LL group demonstrated superior success rates compared to balloon papillary dilation (93.9% vs 72.7%; *P*=0.021). Logistic regression analysis revealed that treatment success was associated with SOC + LL use (OR: 8.7, 95%CI: 1.3-59.3; *P*=0.026), a stone-to-duct ratio ≤1 (OR: 28.8, 95%CI: 1.2-687.6; *P*=0.038), and absence of a narrowed bile duct (OR: 26.9, 95%CI: 1.3-558.2; *P*=0.034)^36^. In a 2018 randomized clinical trial, Franzini et al. compared SOC + EHL with balloon papillary dilation for difficult stones. Initial complete stone clearance rates were 74.5% (77.1% in the SOC group vs 72% in the balloon dilation group, *P*>0.05). After a second session, the overall success rate reached 90.1%. Although no significant differences in technical success, radiation exposure, or adverse events were noted, crossover analysis revealed that rescue therapy with SOC + EHL had a higher success rate (75%) in patients initially treated with balloon dilation compared to rescue balloon dilation in the SOC group (30%)^32^.

Recommendation 3: SOC with EHL or LL is effective and safe for the management of difficult-to-remove bile duct stones.

(Level of evidence: IA; rate of agreement: 94.4%).

Korrapati and colleagues conducted a systematic review and meta-analysis to evaluate the efficacy, accuracy, and adverse events associated with SOC. The review included 49 studies, 33 of which specifically focused on difficult-to-remove stones. The estimated global stone clearance rate (n=31 studies) was 88% (95%CI: 85%-91%), with no significant evidence of heterogeneity (*P*=0.09; I²=26.14). Regarding stone recurrence (n=6 studies), the estimated rate was 13% (95%CI: 7-20%), also with no evidence of heterogeneity (*P*=0.13; I²=40.09) or publication bias (*P*=0.55). The estimated rate of general adverse events was 7% (95%CI: 6-9%), with specific estimates for pancreatitis (2%; 95%CI: 2-3%), cholangitis (4%; 95%CI: 3-5%), perforation (1%; 95%CI: 1-2%), and other adverse events (3%; 95%CI: 2-4%). The rate of severe adverse events was estimated at 1% (95%CI: 1-2%)^37^.

A systematic review conducted by Jin and colleagues investigated the efficacy and safety of SOC in combination with LL or EHL. Complete stone removal was reported in 23 studies, achieving a combined success rate of 94.3% (95%CI: 90.2-97.5%; I²=80%). Additionally, 22 studies reported single-session stone clearance, with a combined rate of 71.1% (95%CI: 62.1-79.5%; I²=89%). Regarding adverse events, 24 studies provided data, with post-ERCP pancreatitis, cholangitis, bleeding, and perforation being the most common complications. Other reported adverse events included fever, abdominal pain, nausea, vomiting, biliary stricture, transient bacteremia, asymptomatic hyperamylasemia, elevated lipase levels, hypotension, hypothermia, ventricular tachycardia, and aspiration pneumonia. The combined adverse event rate was 6.1% (95%CI: 3.8-8.7%; I²=83%)^29^.

Recommendation 4: there is no evidence to support the superiority of EHL or LL when performed during cholangioscopy. The choice between these techniques should be based on equipment availability and the endoscopist’s experience.

(Level of evidence: III; rate of agreement: 94.44%).

Gutierrez and colleagues conducted a multicenter retrospective study evaluating SOC with EHL or LL for the management of difficult-to-remove bile duct stones, including 407 patients (60.4% women, mean age 64.2±18 years)^33^. Successful ductal clearance in a single EHL/LL session was achieved in 315 cases (77.4%). There were no significant differences between EHL and LL regarding technical success (defined as ductal clearance) (96.7% vs 99%, *P*=0.31) or adverse events (3.3% vs 5%, *P*=0.54). Similarly, Bokemeyer and colleagues compared the efficacy of SOC using LL versus EHL in treating bile duct stones and found no significant difference in complete stone clearance rates (LL: 65.9% vs EHL: 67.7%, *P*=0.868)^38^.

Recommendation 5: the longer procedure time for SOC, compared to mechanical lithotripsy or balloon-assisted papillary dilation, is not associated with an increased incidence of adverse events or prolonged radiation exposure.

(Level of evidence: IB; rate of agreement: 83.3%).

In a study comparing mechanical lithotripsy via ERCP and laser lithotripsy with SOC, there were no significant differences in total procedure times (83 vs 66 minutes; *P*=0.23) or stone removal times (53 vs 39 minutes; *P*=0.26). However, the average radiation exposure time (21 vs 11 minutes; *P*<0.01) and the average dose area product (40,745 vs 20,989 mGycm²; *P*=0.04) were significantly higher in the mechanical lithotripsy group compared to the laser lithotripsy group. The adverse event rates were similar between the groups (13% vs 6%; *P*=0.76)^31^.

In another study, Buxbaum and colleagues observed that the average procedure time was significantly longer in the SOC group (120.7 minutes) compared to the conventional therapy group (81.2 minutes; *P*=0.0008). After adjusting for age and comorbidities, this difference remained significant (*P*=0.0014). In the SOC - guided LL group, the average procedure time was 50.0±27.1 minutes, and fluoroscopy times were similar between the groups. The adverse event rates were also similar between the two treatment groups (OR: 0.8; 95%CI: 0.1-5.0)^30^.

Franzini and colleagues reported statistically significant differences in procedure times between the SOC and mechanical lithotripsy groups (72.3 minutes vs 47.1 minutes; difference of -25.2 minutes; 95%CI: -12.48 to -37.91), while radiation exposure times did not differ significantly between the groups^32^.

### Primary sclerosing cholangitis

Recommendation 1: the use of SOC is recommended in patients with Primary Sclerosing Cholangitis (PSC) when there is progression of biliary strictures, elevated serum tumor markers (such as CA19-9), or inconclusive cytological brushing results. SOC is useful for determining the presence of dysplasia or cholangiocarcinoma, providing support for therapeutic decision-making.

(Level of evidence: IIB; rate of agreement: 94.5%).

Patients with PSC have a significantly increased risk of cholangiocarcinoma (CCA). Identifying incidental CCA in these patients before liver transplantation is crucial, as the incidental finding of CCA in the explanted liver significantly increases the mortality rate compared to benign histopathology (HR 16.0; 95%CI, 5.6-45.4; *P*<.001)^39^. A retrospective study conducted in two liver transplant centers in Sweden^39^ ought to identify the most effective strategy for detecting early dysplasia or CCA in PSC patients who are candidates for liver transplantation. A total of 225 patients were included in the study. Cytology via brushing in a single ERCP session showed 57% sensitivity for detecting CCA and dysplasia in the explanted liver. Sensitivity increased to 82% when the CPRE with cytological brushing was repeated and reached 100% when the CPRE was repeated with SOC assistance. The study concluded that repeating cytological brushing, especially when combined with cholangioscopy, is superior to performing a single-session brushing. Similar findings were reported in other observational studies^40^ ^,^ ^41^ and a systematic review^42^, which showed the superiority of SOC compared to traditional methods such as cholangiography and cytological brushing.

A prospective, multicenter clinical trial evaluated the impact of SOC in managing biliary pathologies. Patients with indeterminate biliary strictures (n=48) or primary sclerosing cholangitis with suspected cholangiocarcinoma (PSC; n=13) were included. The study compared planned management before and after SOC and showed changes in management in 69.2% of cases. Sensitivity for detecting malignancy was moderate, but specificity and positive predictive values were high (100%). In the PSC group, although SOC remained inconclusive in nearly one-third of the patients, the proportion of inadequate treatments was reduced by 63%, and planned treatment was modified in more than two-thirds of the patients. Despite limitations such as possible delays in cancer diagnosis, SOC proved effective in reducing unnecessary surgeries and improving diagnostic accuracy^26^.

Another single-center prospective study^43^ also highlighted the role of SOC in managing PSC patients, evaluating 47 patients who underwent SOC. SOC was technically successful in 96% of cases and allowed for adequate sampling in most cases. However, the limited sensitivity of 33% for detecting CCA indicated the need for supplementation with other diagnostic modalities.

Despite its suboptimal performance, SOC with targeted biopsies appears to be the most accurate modality for diagnosing cholangiocarcinoma in PSC patients. A systematic review with meta-analysis assessed the efficacy of various CPRE-based modalities, such as cytological brushing, FISH (fluorescence in situ hybridization), laser confocal endomicroscopy (pCLE), and SOC, in diagnosing CCA in patients with biliary strictures associated with PSC. The authors showed that SOC had the highest diagnostic accuracy (96%), with 65% sensitivity and 97% specificity. The pooled odds ratio for detecting CCA was 59 (95%CI, 10-341), reinforcing the superiority of SOC compared to other techniques^42^. This study supports the findings of Majeed and collaborators, who demonstrated the superiority of combining repeated cytological brushing and SOC in detecting dysplasia or CCA in PSC patients^39^. In both studies, SOC demonstrated greater diagnostic accuracy, particularly due to its ability to perform targeted biopsies in complex strictures. However, technical limitations, such as the difficulty of advancing the cholangioscope in PSC patients and post-procedural complications like cholangitis remain significant challenges^44^. Therefore in this set of patients, SOC should be cautiously considered due to the elevated risk of cholangitis, especially when biliary drainage is incomplete, or strictures are severe.

### Peroral pancreatoscopy

Recommendation 1: POP with lithotripsy is indicated for patients with chronic pancreatitis and complex pancreatic lithiasis who have failed initial treatment with ERCP combined with ESWL [ET/ESWL].

(Level of evidence: III; rate of agreement: 82.4%).

A retrospective study involving 98 patients with chronic pancreatitis and pancreatic lithiasis showed successful stone fragmentation in 74.5% of patients initially treated with ERCP combined with ESWL. In cases where initial therapy failed, the addition of EHL guided by POP or X-ray resulted in success in 50% of patients (7/14). Six patients were treated with outpatient ESWL, all successfully. Successful guidewire passage through the main pancreatic duct (MPD) stricture was identified as a key factor for stone fragmentation success^45^. The study also highlighted that multiple ESWL sessions might be necessary to achieve technical success, suggesting that EHL could be considered for cases where stones persist after 10 sessions^45^.

However, it is important to note that this recommendation is based on a single study, requiring cautious evaluation before routine adoption of this practice. The decision to include EHL-POP as a second-line treatment should be made based on clinical expertise, individual outcomes, and the complexity of the case.

Recommendation 2: For managing patients with pancreatic duct stones and pain associated with chronic pancreatitis, POP with lithotripsy is recommended as an effective alternative to extracorporeal shock wave lithotripsy (ESWL).

(Level of evidence: III; rate of agreement: 94.1%).

A systematic review of case-control and cohort studies compared peroral pancreatoscopy-guided intracorporeal lithotripsy (EHL or LL) with ESWL for treating large stones in the main pancreatic duct. POP showed technical success rates ranging from 43% to 100%, whereas ESWL achieved success rates of 59% to 80%. POP also demonstrated adverse event rates between 0% and 13.5%, making it an effective alternative, particularly for cases where ESWL has failed^46^.

In a multicenter retrospective study of 28 patients with calcified chronic pancreatitis, POP-LL achieved 79% technical success (complete clearance) and 89% clinical improvement, including pain reduction and decreased opioid use. Mild adverse events occurred in 29% of patients. Despite requiring multiple sessions, POP-LL proved to be an effective alternative to ESWL, especially in experienced centers^47^.

A retrospective cohort study of 258 patients compared ESWL and POP, revealing similar technical success rates (86.7% for ESWL and 88.9% for POP). However, POP required fewer procedures (1.6 vs 3.1) and shorter treatment times (101.6 minutes vs 191.8 minutes). While promising, these findings may be limited to specialized centers^48^.

A prospective multicenter study evaluating POP with lithotripsy reported a 90% technical success rate and pain relief in 82.4% of cases. Serious adverse events occurred in 12.5% of patients but were managed conservatively. However, the absence of a direct control group limits interpretation^49^.

In a multicenter study comparing LL-POP and ESWL in 66 patients, LL-POP achieved higher technical success (78.9% vs 70.2%) and required fewer sessions (median of 1 vs 5), although it was associated with a slightly higher complication rate (21% vs 6.3%, *P*=0.09)^50^.

These studies suggest that POP with lithotripsy is an effective alternative to ESWL, with advantages such as a reduced number of procedures. However, results should be interpreted cautiously, considering the requirement for specialized centers and the lack of randomized trials.

Recommendation 3: Peroral pancreatoscopy (POP) with EHL or LL is effective and safe for treating intraductal pancreatic stones in patients with chronic pancreatitis.

(Level of evidence: IB; rate of agreement: 94.1%).

The comparison between electrohydraulic lithotripsy guided by peroral pancreatoscopy (EHL-POP) and laser lithotripsy (LL-POP) for treating pancreatic duct stones in chronic pancreatitis patients shows similar results regarding efficacy and safety, as evidenced by recent studies. McCarty et al. conducted a meta-analysis that found no statistically significant differences between the two techniques in terms of technical success, stone fragmentation, and adverse events. However, LL-POP demonstrated a slightly higher technical success rate (97.74% vs 85.92% for EHL) and a lower adverse event rate (7.09% vs 10.24%)^51^.

These findings are supported by Saghir et al., who analyzed 383 patients and also reported higher technical and clinical success rates for LL (89.3% and 88.2%) compared to EHL (70.3% and 66.5%), although the difference in adverse events was small^52^. Similar results were observed in a study by Guzmán-Calderón et al., who analyzed 15 studies with 370 patients and concluded that both techniques are effective, with very close technical and clinical success rates (EHL: 92.3% and 91.6%; LL: 91.7% and 86.6%)^53^.

While these studies indicate that both techniques are safe and effective options, the results should be interpreted cautiously. The studies were conducted in referral centers and included patients who had not undergone POP as a first-line treatment. Additionally, variations in stone location, size, and prior treatment attempts may influence outcomes.

Therefore, both EHL-POP and LL-POP are recommended for the treatment of pancreatic stones. The choice between them should depend on factors such as operator experience and patient characteristics, as both are supported by robust evidence of their efficacy and safety.

In addition to its use in native pancreatic anatomy, emerging evidence suggests that POP may be feasible and effective in selected patients with surgically altered anatomy. Bonin et al.^54^ reported a successful case of antegrade POP performed after endoscopic ultrasound (EUS)-guided recanalization of a complete pancreaticojejunal stenosis in a patient with a history of pancreaticoduodenectomy. Using a transgastric approach, a digital cholangioscope was advanced into the pancreatic duct, enabling direct visualization and removal of a 4 mm stone without the need for lithotripsy. This case highlights the potential role of antegrade POP in managing pancreatic duct stones in complex postoperative settings, particularly in expert centers. However, as this evidence is based on a single case report, its applicability should be carefully considered.

Recommendation 4: In patients with main-duct intraductal papillary mucinous neoplasms (IPMN), POP can be used to assess tumor extent, aiding in surgical resection.

(Level of evidence: IB; rate of agreement: 100%).

A prospective study involving 41 patients demonstrated that POP was successful in 95% of cases and influenced clinical decision-making in 76%, though the exact nature of its impact was not specified. Additionally, 17% of patients developed post-ERCP pancreatitis, raising concerns about the procedure’s safety, particularly in its early years of application^55^.

A systematic review of 4 retrospective and 1 prospective study, totaling 142 patients undergoing intraoperative pancreatoscopy (IOP), evaluated the technique’s utility in determining the extent of main pancreatic duct involvement in IPMN and guiding the extent of pancreatic resection. IOP detected additional lesions in 48 patients (34%), leading to changes in the surgical plan in 34% of cases. No procedure-related complications were reported. The study concluded that IOP is a safe and effective technique for identifying additional lesions that influence surgical strategy. However, the authors emphasized the heterogeneity and small sample sizes of the included studies, highlighting the need for prospective multicenter trials to clarify IOP’s role and its impact on surgical strategies for IPMN^56^.

These findings suggest that POP, when combined with other techniques, enhances diagnostic accuracy and surgical decision-making in IPMN management. However, while the use of POP in IPMN is promising, some studies suggest a theoretical risk of intraductal tumor cell dissemination^57^ ^,^ ^58^.

### Post-liver transplantation

Recommendation 1: In post-liver transplant patients, SOC is useful for evaluating and treating anastomotic strictures, recanalizing complete strictures, managing refractory strictures, and ensuring precise stent placement.

(Level of evidence: III; rate of agreement: 94.1%).

Despite advances in surgical techniques, biliary complications remain a challenge in liver transplantation. Combining endoscopic retrograde cholangiopancreatography (ERCP) with SOC has shown additional benefits compared to ERCP alone.

A prospective observational study conducted at the University Hospital of Muenster, Germany, involving 26 post-transplant patients, demonstrated that ERCP combined with SOC was more effective in 46.2% of cases. This combination enabled more precise cannulation of strictures and better detection of bile duct stones and casts compared to ERCP alone^59^. Similarly, a prospective study at the Hospital Clinic of Barcelona with 16 patients confirmed these findings, achieving a 93.8% technical success rate in visualizing the biliary tract and collecting biopsies. SOC integrated with ERCP allowed the identification of visual patterns in anastomotic strictures, which could predict treatment responses^60^. Additionally, a case series involving 11 patients with complete anastomotic strictures post-liver transplant demonstrated that scar puncture using the stiff end of a guidewire under direct SOC visualization was successful in 76.9% of cases. This technique proved to be both feasible and safe, particularly when SOC was integrated with ERCP for direct visualization of the strictures^61^.

These studies suggest that combining ERCP with SOC is more effective in addressing post-transplant biliary complications, offering greater diagnostic and therapeutic precision, especially in complex cases.

### Antibiotic prophylaxis recommendation

Recommendation 1: Antibiotic prophylaxis is mandatory for all patients undergoing SOC due to the high risk of cholangitis.

(Level of evidence: III; rate of agreement: 100%).

Most studies reviewed implemented antibiotic prophylaxis for all patients prior to undergoing SOC.^2^ ^,^ ^13^ ^,^ ^15^ ^,^ ^16^ ^,^ ^18^ ^-^ ^20^ ^,^ ^26^ ^,^ ^62^. A multicenter retrospective study reported a cholangitis rate of 1% (n=102) in patients who received peri-procedural antibiotics, compared to 12.8% (n=148) in those who did not (*P*<0.001). Specifically, among 102 procedures where patients received a single antibiotic dose before SOC, only one patient developed cholangitis (1%). In contrast, 19 out of 148 patients who did not receive antibiotics developed cholangitis (12.8%). These findings support the recommendation of peri-procedural antibiotics^63^. Another retrospective study involving 20 centers and 289 patients reported that when antibiotic prophylaxis was administered to all patients, only three cases of cholangitis were observed (1.0%)^25^. Based on the available evidence, a single pre-procedural dose of a third-generation cephalosporin or fluoroquinolone is suggested. Post-procedural continuation should be individualized.

## DISCUSSION

Indeterminate biliary strictures present diagnostic challenges due to unclear causes and inconclusive imaging or biopsies, with a high likelihood of malignancy. Early and accurate identification of malignancy improves survival and avoids unnecessary surgeries for benign conditions. The panel made fully agreement that SOC combined with guided biopsy is the most effective approach for diagnosing indeterminate biliary strictures, offering greater sensitivity than traditional methods like ERCP. The Level of evidence for this recommendation is II, and the main limitations include technical difficulties in evaluating distal biliary strictures and variability in biopsy adequacy. Additionally, operator experience significantly affects the outcomes.

For complex biliary stones, especially those that are large, multiple, or intrahepatic, SOC with EHL or LL is recommended when conventional therapies fail. Studies show that this combination is highly effective, with success rates significantly higher than mechanical lithotripsy. The Level of evidence is II, and the limitations include the extended procedure time required for SOC, which may be considered but does not increase adverse events. Moreover, there is no conclusive evidence indicating that EHL is superior to LL, and the choice depends on available resources and operator experience.

SOC is also beneficial in managing PSC, especially in detecting CCA or dysplasia. It is more accurate than traditional diagnostic methods expediting the tissue diagnosis. The Level of evidence is II, and the limitations include challenges in passing the cholangioscope and the potential need for supplementary diagnostic methods. These issues suggest the need for skilled operators and further research to confirm its universal applicability.

For patients with chronic pancreatitis and intraductal pancreatic stones, POP with lithotripsy is recommended, particularly when ESWL fails. POP shows higher technical success and fewer procedures compared to ESWL, although the evidence is based on specialized center studies. The Level of evidence is III, with the limitation being the lack of randomized controlled trials and the reliance on retrospective studies, which may limit generalizability.

In post-liver transplant patients, combining ERCP with SOC is recommended for biliary complications, offering better diagnostic and therapeutic precision. The Level of evidence is III, and the limitations include the absence of randomized controlled trials, small sample sizes, and potential bias from studies conducted in specialized centers, which may limit the generalizability of the findings.

Antibiotic prophylaxis is mandatory to prevent cholangitis in all patients undergoing SOC, with studies showing a significant reduction in cholangitis rates when antibiotics are used peri-procedurally. The Level of evidence is III, and the limitations include the reliance on retrospective data, potential confounding factors, and the lack of prospective studies specifically evaluating antibiotic prophylaxis in the context of SOC. 

Future expansions of this consensus could include the development of visual clinical decision-making algorithms, particularly for challenging cases such as indeterminate strictures and difficult stones, which are already well addressed here. Additionally, incorporating emerging technologies like artificial intelligence-assisted cholangioscopy could enhance automated image evaluation and diagnostic precision. It would also be valuable to evaluate the learning curve for cholangioscopy and propose formalized training programs, including establishing minimum supervised case requirements for certification. Furthermore, while this consensus does not cover the role of cholangioscopy in advanced malignancies, such as targeted stent placement or biliary decompression in unresectable cholangiocarcinoma, this represents an important area for future investigation. Lastly, consideration of cost-effectiveness and logistical challenges specific to the Brazilian healthcare system will be essential to support widespread implementation.

## CONCLUSION

This consensus represents the first Brazilian recommendations on cholangiopancreatoscopy, providing a framework to standardize and optimize the clinical application of this technology. While the recommendations garnered strong agreement, some of them are grounded in studies with notable limitations, underscoring the potential for refinement as new evidence becomes available. The findings highlight the need for robust, multicenter research to validate these methods and expand their applicability across diverse patient populations, ensuring continuous improvement in clinical outcomes.

## Data Availability

Data-available-upon-request
